# Machinability investigation in turning of high density fiberboard

**DOI:** 10.1371/journal.pone.0203838

**Published:** 2018-09-13

**Authors:** Zhaolong Zhu, Dietrich Buck, Xiaolei Guo, Mats Ekevad, Pingxiang Cao, Zhenzeng Wu

**Affiliations:** 1 College of Material Science and Engineering, Nanjing Forestry University, Nanjing, Jiangsu, China; 2 Division of Wood Science and Engineering, Luleå University of Technology, Skellefteå, Sweden; 3 Department of Material Engineering, Fujian Agriculture and Forestry University, Fujian, China; University of Vigo, SPAIN

## Abstract

A series of experiments were conducted to assess the machinability of high density fiberboard using cemented carbide cutting tools. The objective of this work was to investigate the influence of two cutting parameters, spindle speed and feed per turn, on cutting forces, chip formation and cutting quality. The results are as follows: cutting forces and chip-breaking length decrease with increasing spindle speed and decreasing feed per turn. In contrast, surface roughness increases with decrease of spindle speed and increase in feed per turn. Chips were divided into four categories based on their shape: dust, particle, splinter, and semicontinuous chips. Chip-breaking length had a similar tendency to the variance of cutting forces with respect to average roughness and mean peak-to-valley height: an increase in the variance of cutting forces resulted in increased average roughness and mean peak-to-valley height. Thus, high cutting speed and low feed rate are parameters suitable for high-quality HDF processing and will improve not only machining quality, but production efficiency.

## Introduction

High density fiberboard (HDF), an engineered wood product with a density of 800–1,100 kg/m^3^, is produced from wood fibers by the addition of wax and resin adhesive. HDF panels are formed in a press at high temperature and pressure [1, 2]. Machining of HDF plays a significant role in determining the characteristics of the resulting product, namely its resistance to deformation, strong nail grip, uniform texture, and high dimensional stability [3, 4]. HDF panels are used in different fields for applications such as furniture, flooring, and packaging. In 2016, the sales volume of laminate flooring, an HDF product, was 2.15×10^8^ m^2^ in China, according to statistics from the China Forest Products Industry Association [5].

Cutting tools play a decisive role in the speed and quality of wood processing. However, ordinary tool materials, like high-speed steel and spring steel, are inadequate to satisfy the demands of modern wood processing due to the tannin and adhesive content of wooden products, which shorten tool life via corrosion. What’s more, unlike metal, aluminum, or plastic, wood-based materials tend to be anisotropic and heterogeneous, especially solid wood, which has three main directions: tangential, longitudinal, and radial; this anisotropy severely reduces the lifetime of cutting tools [6], especially ceramic cutting tools with high hardness but poor toughness [7]. Although diamond cutting tools have a sharp edge, with a hardness of 10000 HV, their high price is a negative because it significantly increases the production cost [8].

The most widely used type of cutting tool on the market today are cemented carbide cutting tools. They are primarily made from a large volume fraction of fine grain refractory metal carbide in a metal binder and are popular among the overwhelming majority of industrial users because of their excellent performance, including outstanding wear resistance, heat resistance, corrosion resistance, toughness, and high hardness, even at high cutting temperatures [9, 10].

A review of the literature shows that cutting forces, chip formation and cutting quality are major subjects in cutting studies. The cutting forces in machining medium density fiberboard have been discussed by Dippon et al. [11]; the forces were expressed as functions of tool geometry, cutting constants, and uncut chip area. Their results showed that the pressure exerted by the uncut chip on the rake face is bigger than the friction on the same area. Zhu et al. [12, 13] analyzed the cutting forces acting on the cutting edge when machining HDF using ceramic cutting tools. They showed that the cutting forces grow with increasing cutting distance, decreasing rake angle and increasing wedge angle. The cutting performance of cemented carbide cutting tools in machining three types of wood–plastic composites was examined by Guo et al. [14], who described that chip thickness had the most significant influence on cutting forces, while rake angle and edge radius had less of an influence.

Ohtani et al. [15] researched the chip formation process in the cutting of wooden porous materials, showing that the shear angle of wooden porous materials changed in the range of approximately eighty degrees when measuring the deformations of individual cell walls. A power-type function empirical mathematical model which displayed the chips’ apparent density drops with decreasing cutting speed, increasing feed rate, decreasing steel hardness, and increasing cutting depth was established by Besliu et al. [16]. They used experimental research on chip deformations generated in high speed face milling of tool steel X210Cr12 to develop the model.

Finally, cutting conditions (tool wear, cutting geometry, cutting parameters, etc.) and workpiece properties (species, moisture content, density, etc.) also have a significant influence on cutting quality [17]. The roughness of the machined surface of a wood flour–polyvinyl chloride composite after up-milling using cemented carbide cutting tools was experimentally determined by Guo et al. [18, 19]. Their results showed that cutting quality at high cutting speed is greater than that at a low cutting speed with the same average chip thickness, and the cutting quality improves with a decrease of the sharpness, nose width, and roughness of worn clearance face. Malkoçoğlu et al. [20] used samples of six types of natural wood to study the surface roughness in planing processes. Their results showed that rake angle has a greater impact than feed speed on surface roughness, and the surface roughness of earlywood is higher than that of latewood.

Although extensive research has been focused on the machinability of wood-based material, there is still a dearth of scientific data about the machining properties of HDF turned with cemented carbide cutting tools. HDF is one of the most common furniture-making materials, an application which requires a high-quality surface. Thus, it is necessary to adjust cutting parameters to improve the machining efficiency and production quality of HDF workpieces.

To that end, the objective of this paper was to determine the machinability of HDF in the turning process using cemented carbide cutting tools. Using statistical analysis, the effects of the cutting parameters spindle speed and feed per turn on cutting forces, chip deformation and machined quality were evaluated. We hope the results are appropriate for use as a guideline for wood processing.

## Materials and methods

### Workpiece

HDF used as the test samples in these experiments was supplied by the Power Dekor Group Co. Ltd., China. The samples were formed as discs, 120 mm diameter × 8 mm thickness. They were a 3:7 mixture of poplar (*Populus alba*) and pine (*Pinus spp*) fiber formed by applying 13 wt% of urea formaldehyde resin under conditions of 180–220°C and 7–10 s/mm pressing. Their detailed material properties are shown in Table 1.

**Table 1:** Mechanical and physical properties of HDF workpieces.

Machining workpiece	Modulus of rupture^a^^b^ (MPa)	Modulus of elasticity^a^^b^ (MPa)	Density^a^^b^ (kg/m^3^)	Moisture content (%)
HDF	49.7 (0.19)	4312 (0.03)	861 (0.01)	6.8 (0.01)

^**a**^ Each value is the mean of ten measured values of ten samples

^**b**^ Number in parentheses is the variation coefficient based on ten measurements

### Cutting tools

**Fig 1:**
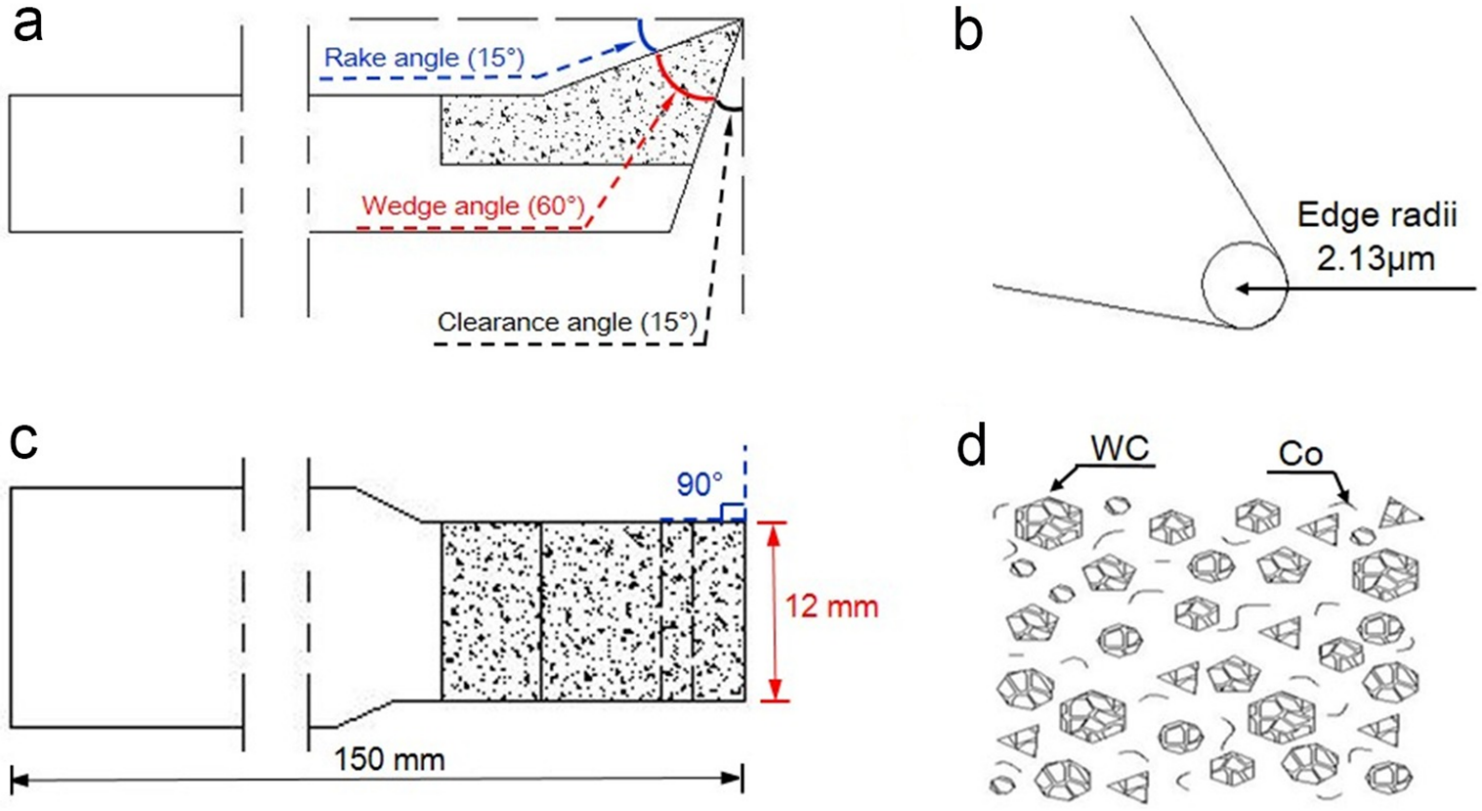
Schematic diagram of the cemented carbide cutting tools. a. cutting angles, b. edge radii, c. dimensions, d. microstructure (WC: tungsten carbide, Co: cobalt).

Specific parameters of the cemented carbide cutting tools are displayed in Fig 1. The blades fixed on the cutter body were manufactured by powder metallurgy using tungsten carbide, cobalt, and other compounds (Leitz Tooling Systems Co. Ltd., China); their chemical composition and mechanical properties are provided in Table 2.

**Table 2:** Chemical composition and mechanical properties of the cemented carbide blades.

Blades	Chemical compositions			Mechanical properties			
Cemented carbide	WC (w%)	Co (w%)	Others (w%)	Thermal conductivity (W/m·K)	Bending strength (GPa)	Hardness (HRA)	Density (g/cm^3^)
	89.6	9.8	0.6	75.36	1.48	88	14.7

### Experimental design

**Fig 2:**
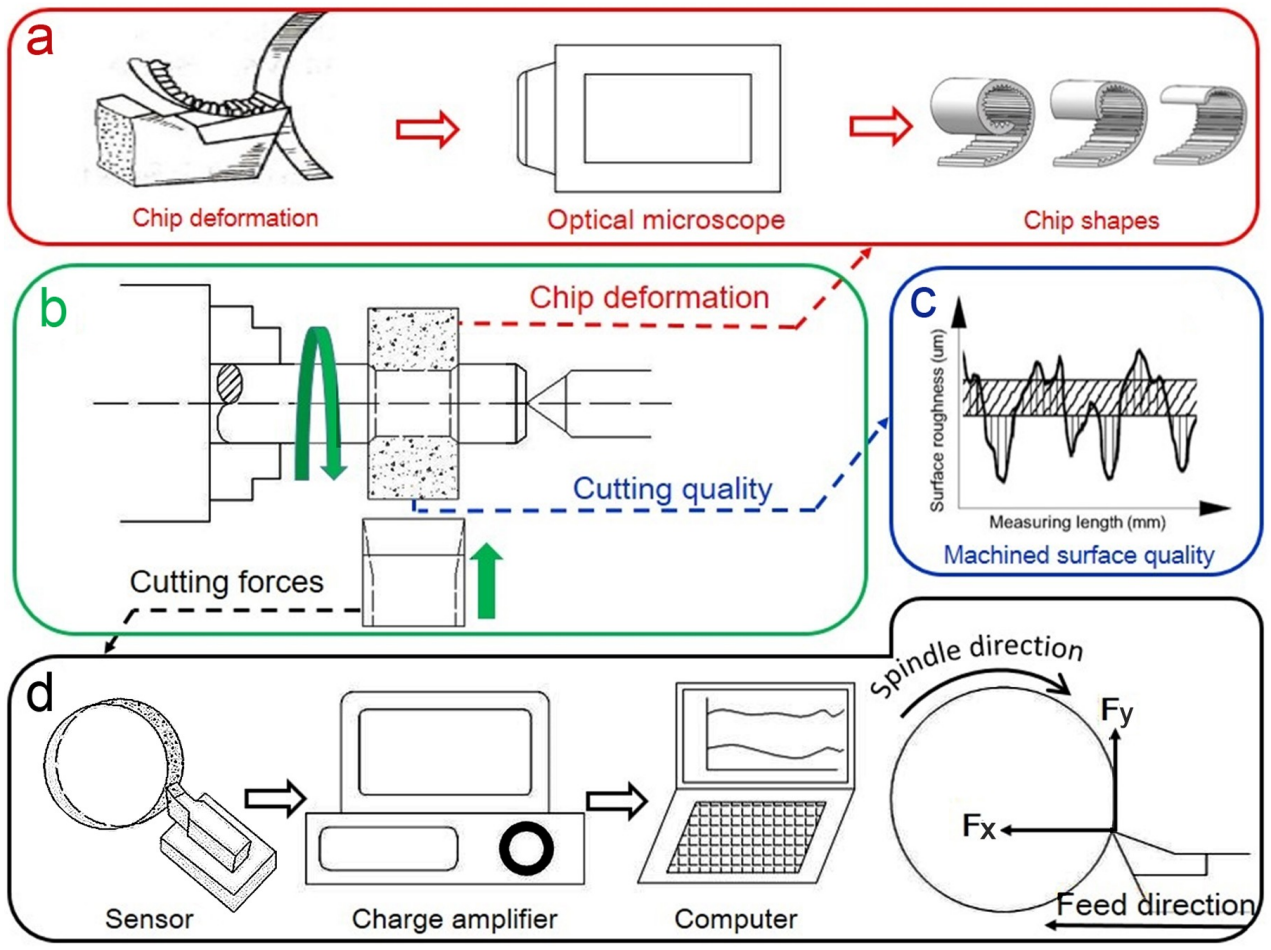
Turning system. a. chip deformation, b. face turning, c. cutting quality, d. cutting forces.

Nine different combinations of cutting parameters were used to evaluate the cutting performance of cemented carbide cutting tools when machining HDF. Each set of cutting parameters was applied and measured five times (Table 3). Experiments were conducted on a commercial lathe by face turning (CA6140, Shenyang Machine Tool Co. Ltd., China), as shown in Fig 2b.

**Table 3:** Turning parameter combinations.

Test number	Spindle speed (n, r/min)	Feed per turn (F_n_, mm/r)
1.1	250	0.1
2	250	0.2
3	250	0.3
4	710	0.1
5	710	0.2
6	710	0.3
7	1120	0.1
8	1120	0.2
9	1120	0.3

As described in Fig 2d, cutting forces exerted by the cutting edge were decomposed into normal force, F_x_, and tangential force, F_y_, which were defined as the component forces parallel and perpendicular to the feed direction, respectively. In this work, a short time constant of 0.5 s was chosen to measure the fast time-variant cutting forces. The dynamic force signals were detected by a sensor (Kistler 9257B, Switzerland) at a rate of 7,100 samples per second and then sent to a charge amplifier (Kistler 5070A, Switzerland), which was used to amplify and condition input data. The mean value and variance of cutting forces at each condition were calculated by the following equations:

$$F_{x}=\sum_{i=1}^{n}x_{i}/\text{n}$$ (1)

$$F_{y}=\sum_{i=1}^{n}y_{i}/\text{n}$$ (2)

$$V_{x}=\sum {\left(f_{x}-F_{X}\right)}^{2}/\text{n}$$ (3)

$$V_{y}={\sum \left(f_{y}-F_{y}\right)}^{2}/\text{n}$$ (4)

where *F*_*x*_ is the mean value of normal force (N); *F*_*y*_ is the mean value of tangential force (N); *f*_*x*_ is the real-time value of normal force (N); *f*_*y*_ is the real-time value of tangential force (N); and *n* is the sample number, 3550.

Meanwhile, different shapes of chips formed from removal HDF material were collected, as can be seen in Fig 2a. The morphology and other characteristics of these chips were observed using an optical microscope (Swift-Duo, Vision Instruments Ltd., England). The breaking lengths of collected chips were also used to evaluate chip formation, which is the length of a broken chip [21].

Finally, after the turning experiment was finished, as presented in Fig 2c, the cutting quality of the machined surface was measured by a surface roughness measuring instrument (S-NEX001SD-12, Vision Instrument Co. Ltd, Japan) and a three-dimensional surface topography profilometer (OLYMPUS DSX510, Japan). Based on the GB/T 1031–2009 and GB/T 131–2006 (ISO 1302:2002), the quality of machined workpiece was evaluated as average roughness (Ra) and mean peak-to-valley high (Rz), which are often important indicators of production quality [18, 19].

## Results and discussion

### Effects of cutting parameters on cutting forces

**Fig 3:**
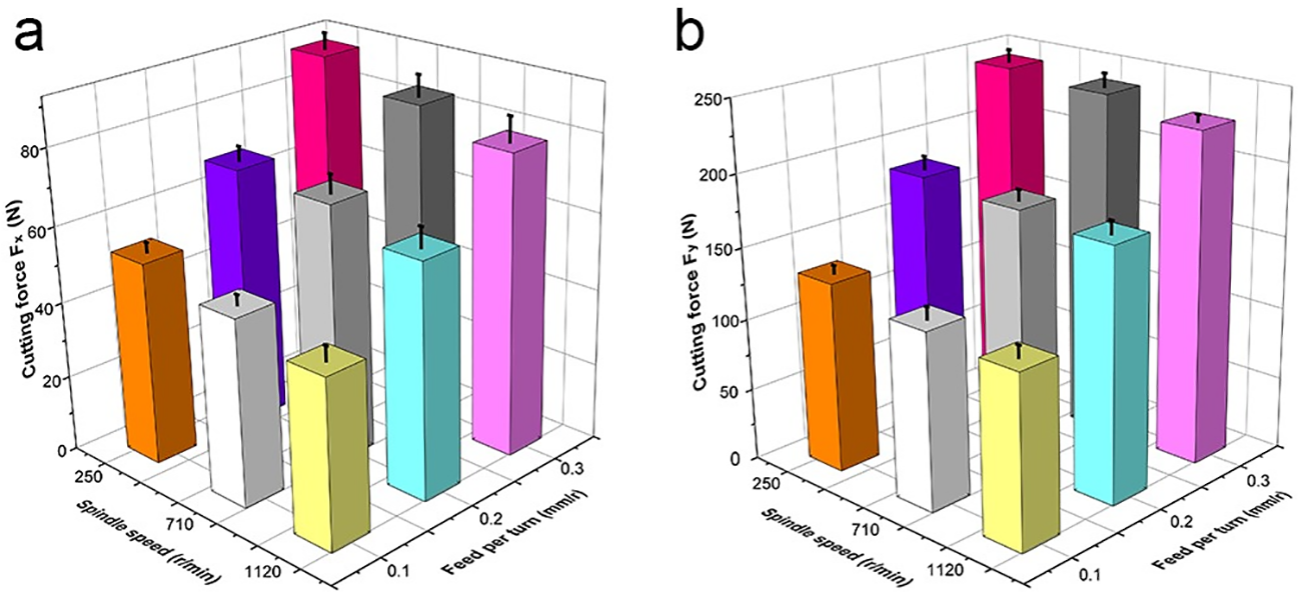
Effects of spindle speed and feed per turn on average cutting forces. a. normal force F_x_, b. tangential force F_y_.

Cutting forces as a function of spindle speed for various feed per turn values are shown in Table 4 and Fig 3. On one hand, both F_x_ and F_y_ decreased with an increase in spindle speed. As the increasing spindle speed raised the cutting temperature between tool face and workpiece, it reduced the strength of HDF and decreased the friction between clearance face and machined HDF surface. On the other hand, cutting forces tended to decrease with a decrease in feed per turn, mainly due to the corresponding reduction in cutting capacity per unit time. With the same spindle speed, decreasing feed per turn reduced the cutting thickness, which decreased the forces acting on the cutting edge due to the removal of less material from workpiece.

**Table 4:** Results of mean cutting forces at different cutting parameters.

Test number	1	2	3	4	5	6	7	8	9
**n (r/min)**	250	250	250	710	710	710	1120	1120	1120
**F**_**n**_ **(mm/r)**	0.1	0.2	0.3	0.1	0.2	0.3	0.1	0.2	0.3
**F**_**x**_ **(N)**	53.2	68.5	90.6	48.8	67.5	84.3	43.9	61.3	78.6
**F**_**y**_ **(N)**	132.2	184.5	243.6	122.7	180.2	239.2	118.6	174.6	228.7

These results are similar to those found in previous research. Changes in cutting force were explored by Zhu et al. [13] in turning HDF by ceramic cutting tools; they also showed that the cutting forces were negative to spindle speed, but positive to feed per turn. In line with those findings, the results of this study suggest that machining parameters of high spindle speed and low feed per turn during HDF processing can decrease energy consumption and improve production efficiency.

### Mechanism of chip formation

**Fig 4:**
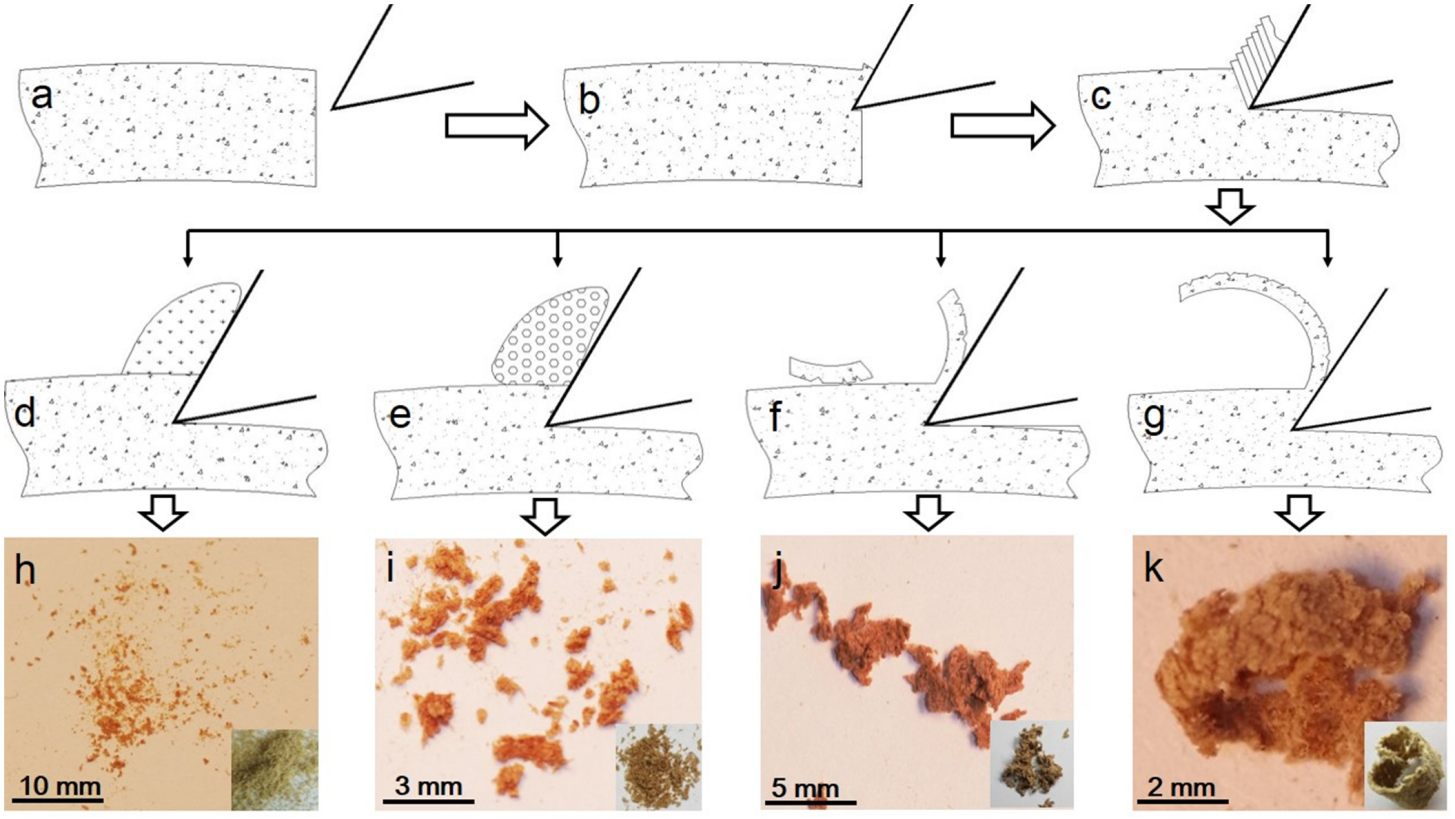
Four chip shapes resulting from different stages of HDF machining. a. The HDF workpiece before turning; b. initial stages of turning; c. shear formation; d. production of dust chip; e. production of particle chip; f. production of splinter chip, g. production of semicontinuous chip; h. dust chips; i. particle chips; j. splinter chips; k. semicontinuous chips.

Chip formation during cutting with different parameters is illustrated in Fig 4. Firstly, the cutting edge sheared the HDF material along the cutting plane (Fig 4a and 4b). Then, the cutting layer of HDF was squeezed by the rake face, and the material removed was formed into chips with different shapes (Fig 4c). According to the experimental results, the chips of HDF were mainly divided into four categories based on their shape: dust chips (Fig 4d and 4h), particle chips (Fig 4e and 4i), splinter chips (Fig 4f and 4j) and semicontinuous chips (Fig 4g and 4k), listed here in order of increasing chip-breaking lengths. During machining of solid wood, five kinds of chips are commonly produced, namely flow, split, compressive, shear and tear chips [22]. Other than the semicontinuous chips of HDF similar to the flow chip of solid wood, the other chip shapes of HDF were different from those produced by machining solid wood.

Dust chips, a powdered form of removed material, had a chip-breaking length of less than 1 mm, and were easily generated at a very small feed per turn of around 0.1 mm/r. In this case, the chip thickness of HDF was small, and the strength was very low. The fiber bundles and adhesive of HDF tended to be crushed into powder form under extrusion from the rake face.

Particle chips, larger than dust chips, had a chip-breaking length of about 2–3 mm. They appeared at a feed per turn slightly higher than 0.1 mm/r or at a high spindle speed of around 1120 r/min. For combinations of cutting parameters which produced particle chips, the extrusion force was greater than the bonding force between fibers, and the removed portions of HDF were sheared and peeled into a small and very loose cluster shape.

Splinter chips, with a chip-breaking length of about 3–5 mm, are mainly formed at moderate spindle speeds of around 710 r/min or moderate feeds per turn, around 0.2 mm/r. For such cutting parameters, the removed HDF material was crushed into flakes, and the produced splinter chips were weakly connected by fiber bundles and adhesive.

Semicontinuous chips with a chip-breaking length of more than 6 mm were produced at large feeds per turn, around 0.3 mm/r, or at low spindle speeds, mainly around 250 r/min. When HDF material was sheared by the cutting edge along the cutting plane, the removed material underwent bending formation under the acting force and was then discharged along the rake face. Semicontinuous chips were shaped into independent chip balls with shapes similar to cylinders. The back side of these chips had many uneven cracks.

### Effects of cutting parameters on chip shape

**Fig 5:**
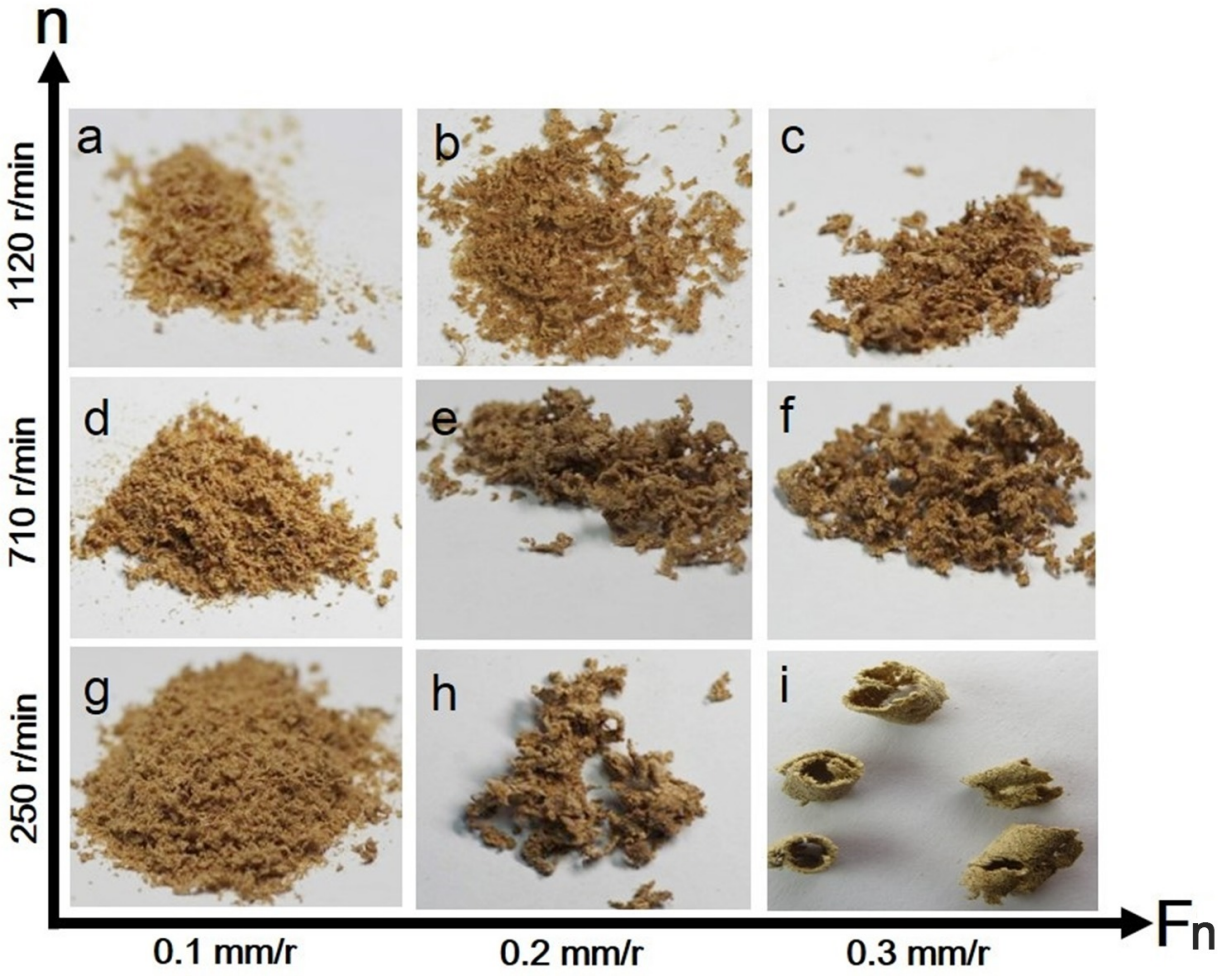
Effects of cutting parameters on chip shapes. a. dust chip; b. particle chip; c. splinter chip; d. dust chip; e. splinter chip; f. splinter chip; g. dust and particle chips; h. splinter chip; i. semicontinuous chip.

The effects of spindle speed and feed per turn on chip deformation are depicted in Fig 5. Firstly, the chip-breaking length decreased with increase of spindle speed for constant feed per turn. For instance, when feed per turn was 0.3 mm/r, the chip shape changed from semicontinuous to splinter to particle with an increase of spindle speed. Secondly, chip-breaking length increased with increasing feed per turn at constant spindle speed; for example, at a spindle speed of 250 r/min, the chip shape went from dust to splinter to semicontinuous with an increase of feed per turn.

### Effects of cutting parameters on surface roughness

**Fig 6:**
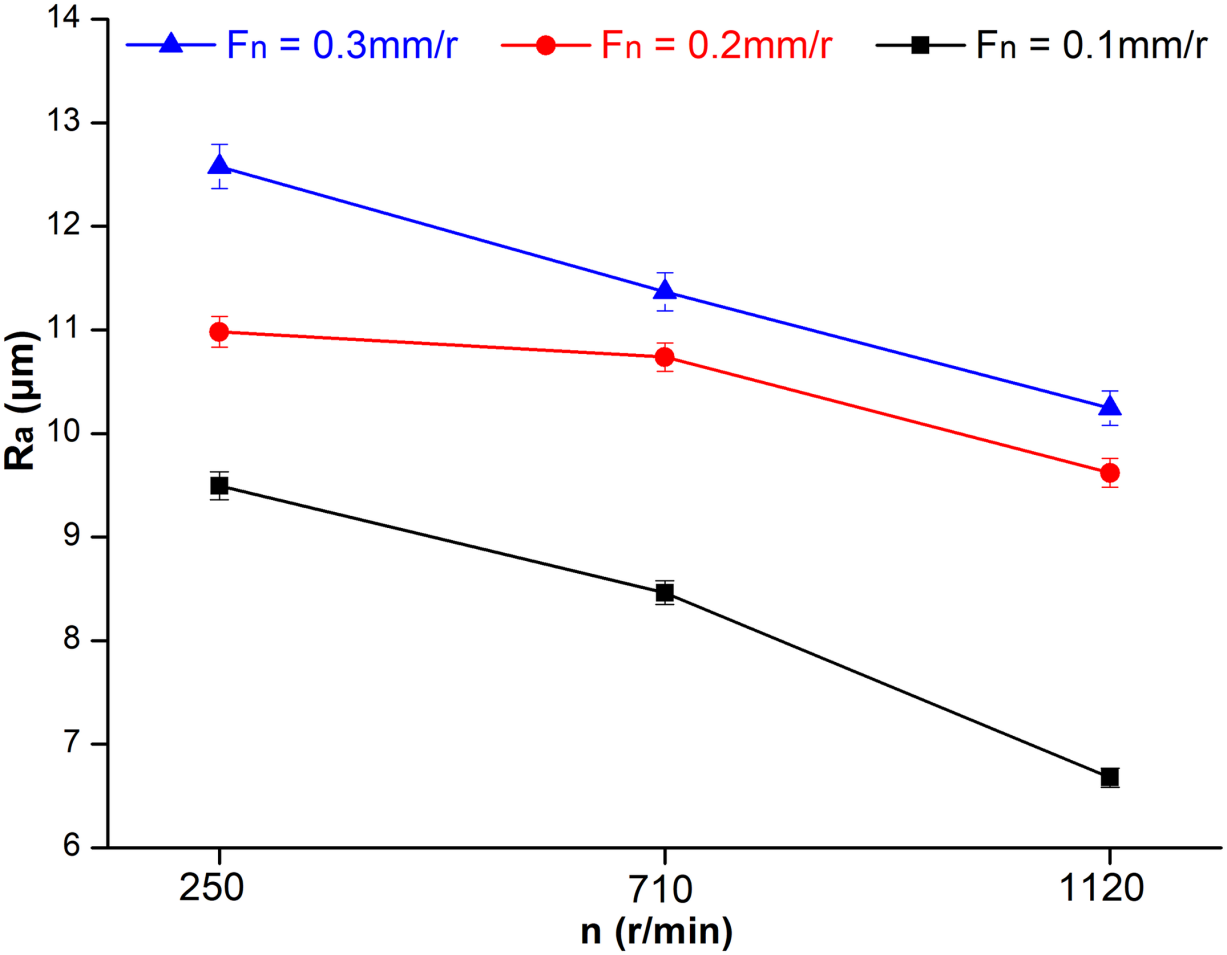
Effects of cutting parameters, spindle speed (n) and feed per turn (F_n_), on surface roughness.

As shown in Table 5 and Fig 6, surface roughness of machined HDF decreased with increase of spindle speed and decrease of feed per turn, and the change in surface roughness was in accord with classical wood cutting theory [23]. With an increase in spindle speed, the cutting edge bit into HDF and the rake face separated the chips from the workpiece more easily, which reduced the surface roughness. However, with increasing feed per turn, the quantity of removed material per unit time was raised, which decreased the cutting stability and increased the surface roughness. Thus, during final machining of HDF, high speed cutting is advised, as it can improve both production efficiency and machining quality.

**Table 5:** Average roughness of machined surface with different cutting parameters.

Test number	1	2	3	4	5	6	7	8	9
**n (r/min)**	250	250	250	710	710	710	1120	1120	1120
**F**_**n**_ **(mm/r)**	0.1	0.2	0.3	0.1	0.2	0.3	0.1	0.2	0.3
**R**_**a**_	9.5	11.0	12.6	8.5	10.7	11.4	6.7	9.6	10.2

### Relation between cutting forces, chip formation and machining quality

**Fig 7:**
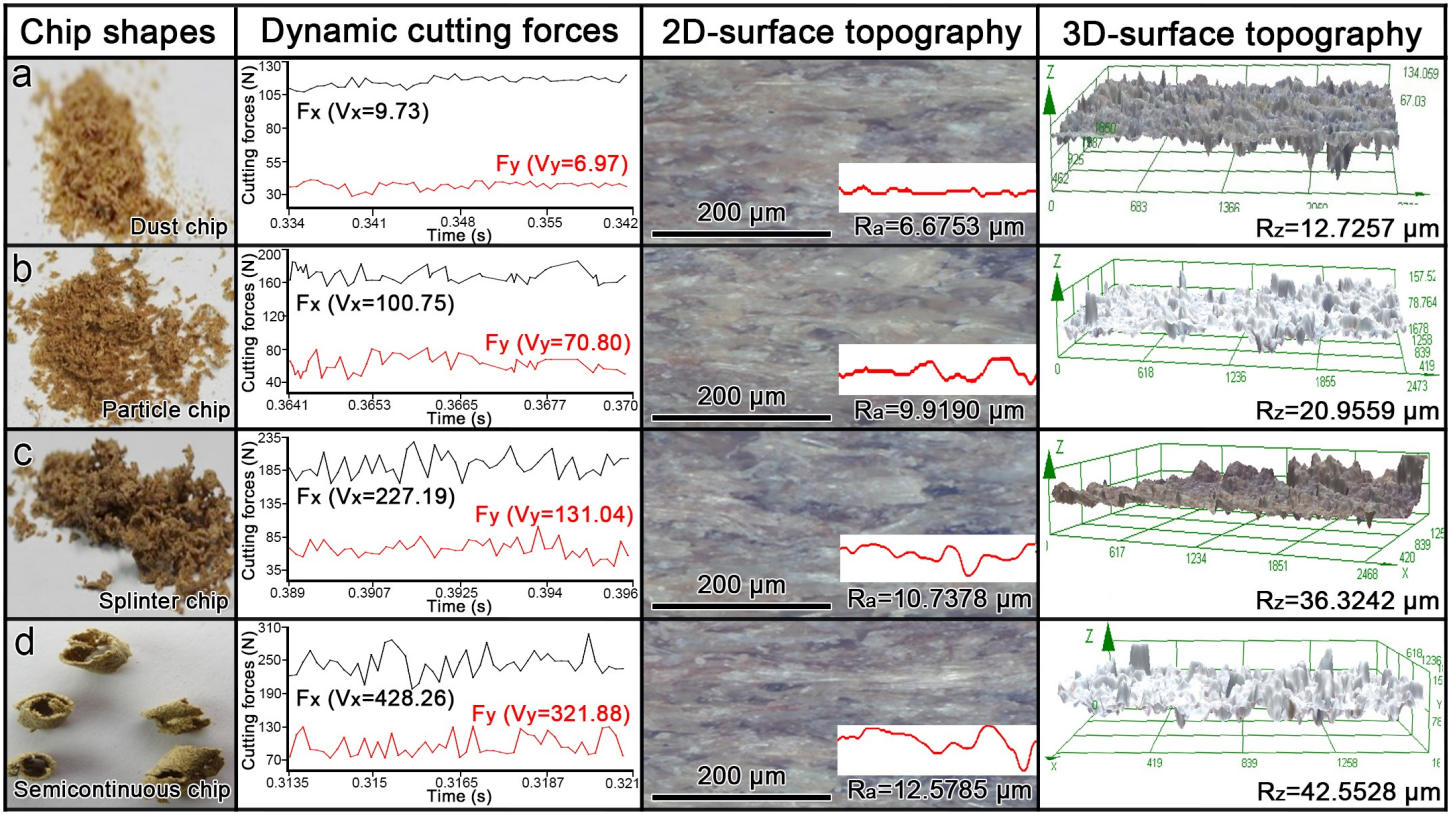
Relation between chip shapes, dynamic cutting forces and machining quality. a. F_n_ = 0.1 mm/r and n = 1120 r/min; b. F_n_ = 0.2 mm/r and n = 1120 r/min; c. F_n_ = 0.2 mm/r and n = 710 r/min; d. F_n_ = 0.3 mm/r and n = 250 r/min. V_x_: variance of cutting force F_x_; V_y_: variance of cutting force Fy; R_a_: average roughness; R_z_: mean peak-to-valley high.

The relation between cutting forces, chip formation and machining quality was investigated (Fig 7). Firstly, when the chips were dust-shaped, the variability of cutting forces was low, and the machined workpiece was very smooth with no obvious pits or peaks on its surface. Then, when particle chips were produced, the variability in cutting forces became larger, and some pits and peaks were observed on the surface, indicating a decrease in machining quality. Thirdly, when splinter chips emerged, the variance of cutting forces became even larger, and more pits and peaks were found. Finally, when semicontinuous chips started to appear, the variance of dynamic cutting forces was largest and the machining quality degraded even further, with many pits and peaks detected on the machined surface by 2D and 3D surface topography.

**Fig 8:**
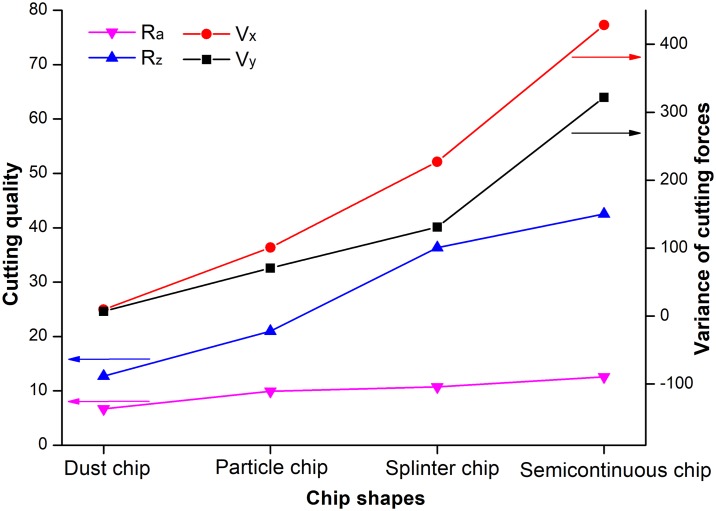
Trends in cutting performance with different chip shapes. R: average roughness; R_z_: mean peak-to-valley high; V_x_: variance of cutting force, F_x_; V_y_: variance of cutting force, F_y_.

In order to research and describe the relation between variables effectively, the variation in trends of cutting performance for different chip shapes was analyzed and is shown in Fig 8. As mentioned above, dust, particle, splinter and semicontinuous chips have increasing chip-breaking lengths, each longer than the one before. Thus, chip-breaking length had a tendency similar to the variance of cutting forces, average roughness and mean peak-to-valley height. In other words, when turning HDF with cemented carbide cutting tools, the shorter the chip-breaking length, the more stable the cutting forces, and the better the resulting machining quality. This result also shows that high-speed cutting and slow feed rates are most suitable for fine processing of HDF. The use of these cutting parameters in a real industrial case will probably improve not only machining quality, but also production efficiency.

## Conclusions

The machinability of HDF was experimentally investigated by turning with cemented carbide cutting tools. The main parameters investigated were cutting forces, chip formation and machining quality. The important findings are summarized as follows:

1. Changes in normal and tangential forces had similar trends: they were all positively related to a decrease in spindle speed, but negatively related to a decrease in feed per turn.
2. Chips produced during machining could be divided into four general categories based on their shapes, namely dust, particle, splinter and semicontinuous chips. Chip-breaking length declined with increase in spindle speed and decrease in feed per turn.
3. The roughness of the machined surface increased with a decrease in spindle speed and an increase in feed per turn.
4. Chip-breaking length had a similar tendency as the variance of cutting forces, with respect to average roughness and mean peak-to-valley height. That is, an increase in chip-breaking length was related to an increase in average roughness and mean peak-to-valley height.

Based on the results, it appears that high cutting speed and low feed rate are suitable choices for fine processing of HDF; these machining conditions not only improve machining quality, but also increase production efficiency.

## References

[1] Garcia RA, Cloutier A, Riedl B. Dimensional stability of MDF panels produced from heat-treated fibers. Holzforschung. 2006;7: 157–184. 10.1515/HF.2006.045

[2] Garcia RA, Cloutier A, Riedl B. Dimensional stability of MDF panels produced from fibers treated with maleated polypropylene wax. Wood Sci Technol. 2005;39: 630–650. 10.1007/s00226-005-0028-7

[3] Chavooshi A, Madhoushi M. Mechanical and physical properties of aluminum powder/MDF dust/polypropylene composites. Constr Build Mater. 2013;44: 214–220. 10.1016/j.conbuildmat.2013.02.079

[4] Madhoushi M, Shahrebabak AB. Mechanical and physical properties of green biocomposite based on medium density fiberboard sanding powder/polyethylene/nanoclay. J Polym Environ. 2016;25: 221–228. 10.1007/s10924-016-0799-y

[5] China Forest Products Industry Association. The sales statistics of floor and decoration paper industry in China in 2016. Chinese Wood Industry. 2017;2: 61 Available from: http://xueshu.baidu.com/s?wd=paperuri%3A%28f1ee48bcf2ab5480535d87f128b8fb98%29&filter=sc_long_sign&tn=SE_xueshusource_2kduw22v&sc_vurl=http%3A%2F%2Fkns.cnki.net%2FKCMS%2Fdetail%2Fdetail.aspx%3Ffilename%3Dmcgy201702021%26dbname%3DCJFD%26dbcode%3DCJFQ&ie=utf-8&sc_us=10977807799113706683

[6] Eyma F, Méausoone PJ, Martin P. Strains and cutting forces involved in the solid wood rotating cutting process. J Mater Process Tech. 2004;148: 220–225. 10.1016/S0924-0136(03)00880-X

[7] Zhu ZL, Guo XL, Na B, Liang XY, Ekevad M, Ji F. Research on cutting performance of ceramic cutting tools in milling high density fiberboard. Wood Res-slovakia. 2017;62: 125–138. Available from: http://www.woodresearch.sk/wr/201701/12.pdf

[8] Sommer F, Talpeanu D, Kern F, Gadow R, Heisel U. Medium density fiberboard machining and wear behavior of injection-molded ceramic composite wood cutting tools. Int J Appl Ceram Tec. 2015;12: 147–156. 10.1111/ijac.12144

[9] Feng WR, Zhou H, Yang SZ. Nano-indentation and wear-resistance behaviors of TiCN films by pulsed plasma on cemented carbide cutting tool. Mat Sci Eng A-struct. 2010;527: 4767–4770. 10.1016/j.msea.2010.04.024

[10] Klaasen H, Kübarsepp J. Wear of advanced cemented carbides for metal forming tool materials. Wear. 2004;256: 846–853. 10.1016/j.wear.2003.08.004

[11] Dippon J, Ren H, Ben AF, Altintas Y. Orthogonal cutting mechanics of medium density fiberboards. Forest Prod J. 2000;50: 25–30. Available from: https://search.proquest.com/docview/214639224?pq-origsite=gscholar

[12] Zhu ZL, Guo XL, Ekevad M, Cao PX, Na B, Zhu NF. The effects of cutting parameters and tool geometry on cutting forces and tool wear in milling high-density fiberboard with ceramic cutting tools. Int J Adv Manuf Tech. 2017;91: 4033–4041. 10.1007/s00170-017-0085-8

[13] Zhu ZL, Guo XL, Zhao F, Qiu XH, Zheng Lin, Cao PX, et al Cutting performance of ceramic tool in turning wood-based composites. J Forest Eng. 2017;2: 119–124.

[14] Guo XL, Ekevad M, Marklund B, Li RR, Cao PX, Crönlund A. Cutting forces and chip morphology during wood plastic composites orthogonal cutting. Bioresources. 2014;9: 2090–2106. doi: 10.15376/biores.9.2.2090-2106

[15] Ohtani T, Komata S. 3423 Chip formation process analyzed by behavior of cellular deformation in cutting of wooden porous material. T Jpn Soc Aeronaut S. 2008;4: 289–290. 10.1299/jsmemecjo.2008.4.0_289

[16] Besliu I, Slatineanu L, Coteața M, Amarandei D. Chip deformation in high speed face milling of the hard tool steel X210CR12. Annals of the University of Oradea. 2014; 1: 1–4. doi: 10.15660/AUOFMTE.2014-1.2955

[17] Aguilera A, Zamora R. Surface roughness in sapwood and heartwood of Blackwood (Acacia melanoxylon R. Br.) machined in 90–0 direction. Eur J Wood Prod. 2009;67, 297–301. 10.1007/s00107-009-0308-2

[18] Guo XL, Ekevad M, Gronlund A, Marklund B, Cao PX. Tool wear and machined surface roughness during wood flour/polyethylene composite using cemented tungsten carbide tools. Bioresources. 2014; 9: 3779–91. doi: 10.15376/biores.9.3.3779-3791

[19] Guo XL, Li RR, Cao PX, Ekevad M, Cristovao L, Marklund B, et al Effect of average chip thickness and cutting speed on cutting forces and surface roughness during peripheral up milling of wood flour/polyvinyl chloride composite. Wood Res-slovakia. 2015; 60: 147–156. Available from: http://www.centrumdp.sk/wr/201501/14.pdf

[20] Malkocoglu A. Machining properties and surface roughness of various wood species planed in different conditions. Build Environ. 2007; 42: 2562–67. 10.1016/j.buildenv.2006.08.028

[21] Wu MY. Research on mechanism and experimental of chip breaking during high pressure cooling turning of superalloys with PCBN tool. J Mech E. 2017; 53: 187 10.3901/JME.2017.09.187

[22] McKenzie W.M. (1961) Fundamental analysis of the wood-cutting process. Nippon Nogeikagaku Kaishi. 42:152–157. Available from: http://hdl.handle.net/2027.42/6536

[23] Koch P. Wood machining processes. New York: The Ronald Press Company, 1964.

